# Introducing the importance and difficulties of a three-step approach to improve nonadherence to antihypertensive drugs: a case series

**DOI:** 10.1097/HJH.0000000000003001

**Published:** 2021-08-23

**Authors:** Laura E.J. Peeters, Jeroen B. van der Net, Kathy Schoenmakers-Buis, Irene M. van der Meer, Emma K. Massey, Liset van Dijk, Teun van Gelder, Birgit C.P. Koch, Jorie Versmissen

**Affiliations:** a Department of Hospital Pharmacy; b Department of Internal Medicine, Erasmus MC, University Medical Centre Rotterdam; c Department of Internal Medicine, Albert Schweitzer Hospital, Dordrecht; d Department of Internal Medicine, HAGA Hospital, The Hague; e Department Pharmaceutical Care, Netherlands Institute for Health Services Research, Utrecht, The Netherlands

**Keywords:** adherence, communication tool, hypertension, intervention, therapeutic drug monitoring

## Abstract

Nonadherence to antihypertensive drugs is an important reason for not reaching blood pressure goals. A possible method to improve nonadherence involves three essential steps: identification of nonadherent patients (step 1), determination of underlying causes (step 2) and a personalized solution (step 3). We present three unique cases to show the importance and difficulties of this three-step approach.

Patients participated in a randomized controlled trial to improve nonadherence to antihypertensive drugs (RHYME-RCT, Dutch Trial Register NL6736). Drug level measurements were used to identify nonadherence to antihypertensive drugs and communication on drug levels was supported by a tailored feedback tool in these patients. These cases showed that a three-step approach of identifying nonadherence and determination of the underlying cause, can lead to a personalized solution to improve therapy even when nonadherence was excluded. Open communication with patients remains an essential part when improving nonadherence.

## INTRODUCTION

Nonadherence to antihypertensive drugs is an important reason for not reaching blood pressure goals, which in turn will lead to an increased risk of cardiovascular disease, stroke and kidney failure [1]. Especially patients that fit the criteria for resistant hypertension are at risk of these consequences because of an uncontrolled blood pressure despite the use of three or more antihypertensive drugs [1].

Improving nonadherence is challenging as the ability of healthcare providers to recognize nonadherent patients is limited [2]. Recognition of these patients can be improved by several identification methods [3]. Although identification of nonadherence is an important step towards improvement of drug therapy, follow-up steps are needed to promote adherence behaviour. Therefore, a three-step approach for improvement of adherence was suggested [4]. In this study, we aimed to apply this approach to antihypertensive drug therapy in the resistant hypertension population.

Step 1 is the identification of nonadherent patients, step 2 is determination of the underlying cause and step 3 is finding a personalized solution to solve the issues that lead to suboptimal drug treatment.

For the identification step, objective methods to assess adherence are preferred to avoid overestimation of the adherence rate. One of the most objective and reliable methods is measurement of drug levels in body fluids. The absence of drug will identify nonadherent patients [3,5].

The next step included sharing information on the absence of drug in blood measured in step 1 combined with the identification of barriers that lead to nonadherence [6]. These barriers can be either practical, such as a drug regime that is difficult to implement in daily life, or perceptual, such as doubts about safety or efficacy of medication or the consequences of the disease [7]. This diversity in causes was also recognized by several models like the WHO adherence model and practical and perceptual barriers to successful medication intake behaviour typology (PPB-typology) [7,8]. Identification of possible barriers is necessary to continue to the third and final step where a personalized solution has to be found to improve nonadherence. A one-size-fits-all approach is a difficult determinant of nonadherence, specific to the individual.

## CASE REPORTS

The three-step approach to improve nonadherence to antihypertensive drugs was applied in a single-blinded randomized controlled trial RHYME-RCT (Resistant Hypertension: Measure to ReaCh Targets, Dutch trial register NL6736). To illustrate the importance and difficulties that can arise using this three-step approach we present three unique patients who participated in this trial. We not only discuss the successes of this approach but also the limitations including a case where expected nonadherence was found to be nonexistent.

**FIGURE 1:**
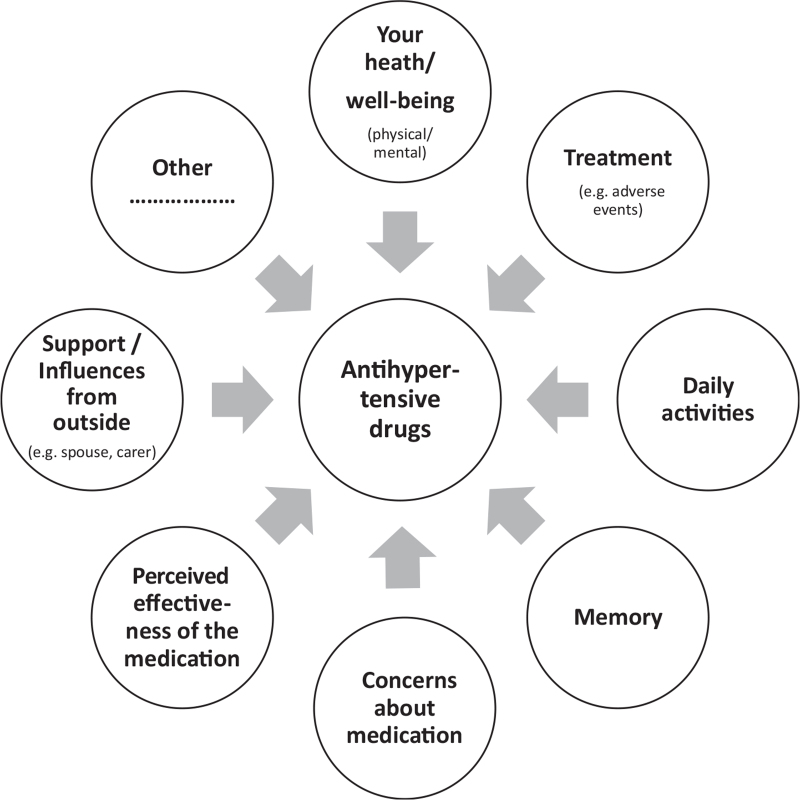
Communication tool to identify practical and perceptual barriers that underlie nonadherence to antihypertensive drugs.

The presented cases were all included in the RHYME-RCT trial and fulfilled the criteria of resistant hypertension. Written informed consent was obtained before participation in the trial. Nonadherence was measured by means of a newly developed blood sampling method called dried blood spot (DBS) to assess the drug concentrations of eight antihypertensive drugs by a finger prick, to allow blood sampling at the same time as blood pressure measurement [9]. A DBS and 24-h ambulatory blood pressure measurement were simultaneously performed at baseline, 3, 6 and 12 months of follow-up. In the control arm, the drug levels were not reported to the treating physician. In the experimental arm, the presence or absence of drugs in blood were reported to the treating physician who then discussed this with the patient in a nonaccusatory way supported by a tailored communication tool based on the previously mentioned WHO model and PPB-typology (Fig. 1) [7,8]. This nonaccusing approach is important to retain the doctor–patient relationship and to create an open environment to discuss possible nonadherence [10]. All participating physicians and specialist nurses in the study followed a 3 h training to carry out this feedback.

### Patient A

This patient is a 53-year-old woman with a history of hypertension for which she had been on treatment in a tertiary hospital for the last 8 years. At the time of inclusion, she was on drug treatment with nifedipine retard 30 mg once daily, spironolactone 12.5 mg once daily, bisoprolol 10 mg once daily and amlodipine 10 mg, valsartan 320 mg and hydrochlorothiazide 25 mg in a combination tablet once daily. Blood pressure remained uncontrolled despite adding drugs and increasing dosages over the years.

At the first study visit, daytime blood pressure was 155/108 mmHg and DBS measurements revealed the absence of amlodipine, valsartan and hydrochlorothiazide (Table 1). During the feedback session, the patient revealed that she did not take the combination tablet every day because of vertigo problems after intake. She had experienced these problems from treatment initiation but was apprehensive to share these side-effects with her treating physician. In this case, the side-effects were identified as barrier for not taking the medication (Fig. 1).

**TABLE 1:** Outcome of drug levels measured by means of a dried blood spot sampling method combined with ultra-high-performance liquid chromatography-tandem mass spectrometry to identify nonadherence to antihypertensive drugs

	Used antihypertensive drugs	Measurement 1	Measurement 2 (3 months follow-up)	Measurement 3 (6 months follow-up)
Patient A				
Blood pressure (mmHg) – average daytime ABPM		155/108	141/100	134/91
Time between intake and sampling (h)		21.0	3.3	3.3
Measured antihypertensive drugs and outcome measured with UPLC-MS/MS (absence of drug in blood = negative)	Amlodipine^a^ Valsartan^a^ Hydrochlorothiazide^a^ Spironolactone Nifedipine Losartan	Negative Negative Negative Positive Positive –	– – Positive Positive Positive Positive	– – Positive Positive Positive Positive
Patient B				
Blood pressure (mmHg) – average daytime ABPM		163/107		
Time between intake and sampling (h)		2.0 (nifedipine) 22.0		
Measured antihypertensive drugs and outcome measured with UPLC-MS/MS (absence of drug in blood = negative)	Irbesartan^a^ Hydrochlorothiazide^a^ Spironolactone Nifedipine	Negative Negative Negative Negative	Lost to follow-up	Lost to follow-up
Patient C				
Blood pressure (mmHg) – average daytime ABPM, heartrate (BPM)		168/101, 95	128/101, 97	139/83, 88
Time between intake and sampling (h)		16.5	7.0	1.0
Measured antihypertensive drugs and outcome measured with UPLC-MS/MS (absence of drug in blood = negative)	Hydrochlorothiazide^a^ Losartan Metoprolol Amiloride^a^	Positive Positive Negative –	Positive Positive Negative Positive	Positive Positive Negative Positive

ABPM, ambulatory blood pressure measurement; BPM, beats per minute; UPLC-MS/MS, ultra-high-performance liquid chromatography-tandem mass spectrometry.

a Used in a single pill combination.

After being made aware of this barrier, the physician switched valsartan to losartan and stopped amlodipine. This resulted in a daytime blood pressure of 141/100 mmHg at 3 months follow-up with adherence for all the medications. After this visit, the dose of nifedipine was increased to 60 mg once daily. At 6 months follow-up, daytime blood pressure improved further to 134/91 mmHg and DBS results showed that she was still adherent to the antihypertensive drug therapy and did not experience side-effects.

### Patient B

This patient is a 40-year-old woman treated with antihypertensive drugs after a diagnosis of hypertension 4 years ago. At the time of inclusion, she used irbesartan 300 mg combined with hydrochlorothiazide 25 mg once daily, nifedipine 60 mg twice daily and spironolactone 50 mg once daily. Nonadherence to medication was suspected but always denied by the patient. Communication was complicated because of a language barrier. She was able to understand and speak Dutch but this was not her native language.

At the first study visit, daytime blood pressure was 163/107 mmHg and none of the antihypertensive drugs were detected in her blood (Table 1). At the intervention appointment, an independent translator was asked to translate. When the patient was told about the negative drug levels, she got very upset and angry. After careful questioning by a nurse specialist and applying the communication tool, she revealed that she had many concerns about her medication and was scared of the possible negative long-term effects of the antihypertensive drugs, including having a stroke or kidney damage. Furthermore, she was worried about her hypertension and rated her personal health at that time with a 1 on a scale from 1 to 10.

The identified barrier was concerns about medication (Fig. 1). Because of these concerns, the conversation focused on reassurance and explanation on the mechanism of action of the medication and development of adverse events. The follow-up plan was to re-evaluate all medication and try to find the most optimal drug treatment for the patient. Unfortunately, the patient failed to show up at any of the follow-up appointments in the hospital. Therefore, the general practitioner was informed about the situation and asked to continue the follow-up.

### Patient C

This patient is a 69-year-old man who was included in the study with an office blood pressure of 192/100 mmHg. At the time of the inclusion, his antihypertensive medication consisted of losartan 100 mg once daily, hydrochlorothiazide 12.5 mg once daily and metoprolol 25 mg once daily. Although the hypertension did not fulfil the definition of resistant hypertension, the patient was included, given the extremely high blood pressure despite three agents including a diuretic. At the first study visit, daytime blood pressure was 168/101 mmHg and the absence of metoprolol in blood was identified (Table 1).

When the treating physician discussed the absence of metoprolol in blood with the patient, he claimed he was adherent to all antihypertensive drugs including metoprolol and did not understand why it could not be detected in his blood. In this case, nonadherence was not confirmed by the patient (Table 1).

At the next measurements at 3 and 6 months, the results remained the same (Table 1). As all other antihypertensive drugs were present in blood and the patient's reported adherence, other explanations for the findings were sought. Therefore, it was suggested to investigate enzymes that were involved in metoprolol metabolism with focus on CYP2D6. A pharmacogenetic analysis showed that the patient was a slow metabolizer of CYP2C9, genotype ∗2/∗11 and an ultra-rapid metabolizer of CYP2D6, genotype ∗1/∗2(xN) with more than three duplicates. This could explain the absence of metoprolol levels in blood and suboptimal blood pressure control.

As a result of the pharmacogenetic analysis, metoprolol was stopped. Meanwhile, amiloride was added. After another 6 months daytime blood pressure decreased to 134/79 mmHg and the patient remained adherent.

## DISCUSSION

We discuss three cases that illustrate the importance of a three-step approach to improve drug therapy in patients with resistance hypertension including identification of nonadherence, determination of the underlying cause and finding a personalized solution. This is the first time that an objective identification method like measuring drug levels in blood by means of a DBS sampling method, is combined with an intervention to understand the underlying barriers of nonadherence and simultaneously addresses these barriers in patients with resistance hypertension [6]. The results from this identification method are essential to initiate conversation with the patient. Furthermore, open communication is key to interpret drug level outcomes and to determine if and why a patient is nonadherent [7]. However, even when using this intervention with very careful communication, improvement of drug therapy can be complicated by insufficient communication skills of healthcare providers or a language barrier.

Our first case (case A) showed that an adverse reaction was the main cause of nonadherence, leading to nonadherence to three drugs from different drug classes. It also revealed the importance of the patient–physician relationship to provide an open and honest environment to discuss problems with medication [10]. After identification of the barrier for adherence, the problem was solved by a switch in medication, which improved blood pressure.

Not only adverse events can lead to nonadherence but also the beliefs about medicine can be an important factor, as illustrated by case B. We postulate that this patient had little trust in the healthcare providers, which, in combination with the negative attitude toward medication, language barrier and absence of a trusted translator, resulted in a complete disengagement of the patient, and therefore, an undesirable outcome. In such unique cases, outreaching strategies are necessary like extra training to deal with these specific problems or referral to a psychologist.

Finally, even when nonadherence is measured with an identification tool, careful interpretation and verification with the patient is needed to prevent false conclusions as our last presented case proved. For this, partnership with the patient and trust of the patient are essential. In case of measuring drug levels, knowledge on the limitations of the method and pharmacokinetics properties of drugs including influencing factors, such as pharmacogenetics is needed to interpret results. For instance, the time of intake and sampling and the dose of the drug should always be taken into account whenever interpreting drug levels for nonadherence. Furthermore, measuring drug levels will give information on the adherence rate at the time of sampling, and therefore, a single measurement cannot be used to determine nonadherence over a longer period of time.

Knowledge of pharmacokinetics, dose and time of intake were needed to eliminate nonadherence in case C and led to the discovery of an ultra-rapid phenotype of CYP2D6. This phenotype resulted in a rapid degradation of metoprolol in blood and subsequently drug levels below the lower limit of detection of the method. To solve this problem with metoprolol, a switch to another beta blocker was recommended.

In conclusion, these cases showed that a three-step approach of identifying nonadherence and determination of the underlying cause, not only gives more insight into the reasons for resistant hypertension but also can lead to a personalized solution to improve antihypertensive drug therapy, even when nonadherence was excluded. Open communication and partnership with patients remains an essential part when improving nonadherence.

## ACKNOWLEDGEMENTS

Funding: This study was supported by a ZonMW grant for Rational Pharmacotherapy.

### Conflicts of interest

L.E.J.P. received lecture fees from Astellas Pharma. The other authors declare that they have no financial relationships to the article and no conflicts of interest.
